# Clinical Utility of the Serum Level of Lipoprotein-Related Phospholipase A2 in Acute Ischemic Stroke With Cerebral Artery Stenosis

**DOI:** 10.3389/fneur.2021.642483

**Published:** 2021-03-04

**Authors:** Jing Cao, Ping Yan, Yajun Zhou, Xia Zhou, Zhongwu Sun, Xiao-Qun Zhu

**Affiliations:** Department of Neurology, The First Affiliated Hospital of Anhui Medical University, Hefei, China

**Keywords:** cerebral artery stenosis, plaque stability, neurologic injury, lipoprotein-associated phospholipase A2, acute ischemic stroke

## Abstract

We aimed to study the clinical utility of serum lipoprotein-associated phospholipase A2 (Lp-PLA2) in acute ischemic stroke (AIS) with cerebral artery stenosis (CAS). We included 200 AIS patients and 90 healthy controls in this study. AIS patients were classified into three subgroups depending on the severity of CAS. They were also classified based on the stability of the carotid plaques. Spearman correlation analysis was performed to determine the correlation relationship between the level of Lp-PLA2 and neurologic injury. Binary logistic regression analysis was performed to determine the independent risk factors for AIS. Receiver operating characteristic (ROC) analysis was performed to assess the diagnostic value of Lp-PLA2 for AIS and for the degree of CAS. We found that the serum level of Lp-PLA2 in AIS patients was significantly higher than that in the control group. Lp-PLA2 was further identified as an independent risk factor for AIS (*p* = 0.001, OR = 1.057). In addition, serum Lp-PLA2 level was the highest in AIS patients with severe CAS or occlusion. Lp-PLA2 level was higher in AIS patients with unstable plaques and in AIS patients with moderate to severe neurological injury. Lp-PLA2 level was positively correlated with National Institutes of Health Stroke Scale (NIHSS) score (*r* = 0.335, *p* = 0.001). We found that the optimal cut-off value for Lp-PLA2 level was 123.365 ng/ml, at which the sensitivity and specificity for the diagnosis of ACI were 74.5 and 86.7%, respectively, and the area under ROC curve (AUC) was 0.892. Similarly, the optimal value for Lp-PLA2 level was 136.46 ng/ml, at which the sensitivity and specificity for the diagnosis of the presence of moderate to severe artery stenosis or occlusion were 79.6 and 95.2%, respectively, and the AUC was 0.938. The ROC curve indicated that serum Lp-PLA2 level has an excellent diagnostic value for AIS and severe stenosis. Based on these results we conclude that Lp-PLA2 could be a potential biomarker to complement the current imaging methods in the prediction and diagnosis of AIS. An elevated Lp-PLA2 level is also correlated with carotid plaque instability, severe neurological injury and cerebrovascular stenosis. Future longitudinal studies are needed to determine whether there is a causative relationship between Lp-PLA2 and AIS.

## Introduction

Acute ischemic stroke (AIS) has a high incidence, disability, mortality, and recurrence rate, which often places a tremendous pain and burden on the patient, the family and the society (1). Intracranial atherosclerotic stenosis is the most common cause of ischemic stroke worldwide (2). Atherosclerotic plaque formation is an early pathological feature of carotid lesions, which can gradually develop into vascular stenosis and may completely block the cerebral blood flow, leading to AIS (3, 4). The role of atherosclerosis as an independent risk factor in AIS has been extensively studied.

Abundant evidence has been provided that inflammation played an important role in the formation and development of atherosclerosis (5). Lp-PLA2, also known as platelet-activating factor acetyl hydrolase, is a phospholipase A2 enzyme that is mainly secreted by macrophages and lymphocytes in atherosclerotic plaques. About 80% of the Lp-PLA2 is bound to the low-density lipoproteins (LDLs) (6). LDLs become oxidized low-density lipoproteins (ox-LDL) by multiple mechanisms after accumulating in the subintima of arterial wall. Lp-PLA2 specifically hydrolyzes the oxidized phosphatidylcholine in ox-LDL. The generated pro-inflammatory products (oxidized free fatty acids and lysophosphatidylcholine) are implicated in endothelial dysfunction and plaque inflammation, which accounts for the proatherogenic property of Lp-PLA2 (6, 7). As a new vascular-specific inflammatory factor, Lp-PLA2 can be broadly involved in the progression of atherosclerosis, including the formation, development and rupture of the plaque (8). Elevated Lp-PLA2 levels are strongly associated with atherosclerosis-related diseases, including heart disease and ischemic stroke (9, 10). In addition, the elevated Lp-PLA2 levels may contribute toward both stroke occurrence and recurrence (11). However, there are few studies on the predictive value of Lp-PLA2 levels to assess the degree of cerebrovascular stenosis and neurological impairment in AIS. The aim of this study was thus to analyze the role of Lp-PLA2 in the acute ischemic stroke with cerebral artery stenosis and provide guidance for clinical application of this biomarker.

## Materials and Methods

### Study Population

Ethics: This study is a retrospective analysis. The ethics review and informed consent was exempted. The study protocol conforms to the ethical guidelines of the 1975 Declaration of Helsinki.

From April 2018 to April 2020 a total of 200 hospitalized patients diagnosed with AIS through head magnetic resonance imaging (MRI) in the Department of Neurology of First Affiliated Hospital of Anhui Medical University were included in this study, including 129 males and 71 females with the age of 50 to 80 years old. Additionally, 90 gender-and age-matched healthy controls were recruited, including 50 males and 40 females with the same age range. All patients were admitted within 72 h of the onset of cerebral infarction. Head MRI, computed tomography angiography (CTA) examination of the head and the neck, and carotid color doppler ultra-sonography were performed for all patients.

The inclusion criteria for the case group were as follows: diagnosis meeting the ACI diagnostic criteria as defined by the Chinese Guidelines for Diagnosis and Treatment of Acute Ischemic Stroke 2018 (12); neurological examination supported the diagnosis of acute cerebral infraction; diagnosis performed within 72 h of the onset and confirmed by head MRI; and that the brain MRI and CTA examinations of patients showed that the fresh infarct lesion was consistent with the anatomical location of cerebral artery stenosis indicating a symptomatic stenosis.

The exclusion criteria for the case group were as follows: onset time beyond 72 h; diagnosis of transient ischemic attack, intracranial hemorrhage, venous sinus thrombosis, or other cerebrovascular diseases; presence of cardiovascular diseases such as atrial fibrillation, valvular heart disease, patent foramen ovale; presence of severe liver and kidney dysfunction, presence of systemic or central nervous system infection, presence of diseases in the hematopoietic system or the immune system, or cancer; allergy to iodine contrast agents; and treatment with statins, hormone, or immunosuppressant drug within 4 weeks before admission.

### Demographic Data Collection

The following patient records were obtained within the first 24 h on admission: gender, age, medical history of vascular risk factors (smoking, alcohol drinking, diastolic blood pressure, systolic blood pressure hypertension, hyperlipidemia, diabetes mellitus, cardiac disease), and history of liver and kidney diseases. Severity of neurological impairment was measured using the NIHSS score, a 15-item neurological evaluation (13). The NIHSS score ranges from 0 to 42 points. The level of patient score was positively correlated with the degree of neurological impairment. As in a previous study (14), patients were divided into the mild neurological injure group (NIHSS <5 points) and the moderate to severe neurological injury group (NIHSS≥5 points).

### Blood Collection and Laboratory Test

Peripheral fasting blood (3 ml) was collected within 48 h after admission for clinical management. The serum was prepared by centrifuging the uncoagulated blood at 3,000 r/min for 10 min and allocated before being stored at −80°C for analysis. Serum Lp-PLA2 level was determined by enzyme linked immunosorbent assay (ELISA) with a commercial kit (Lp-PLA2 quantitative assay kit, Tianjin Kangerke Biotechnology Co. Ltd., China). The normal range of the serum Lp-PLA2 level was defined as 0 to 170 ng/ml.

Other blood chemistry parameters, including the levels of fasting blood glucose (Glu), creatinine (CRE), uric acid (UA), total cholesterol (TC), triacylglycerol (TG), high density lipoprotein cholesterol (HDL-C), low density lipoprotein cholesterol (LDL-C) and other blood lipid indicators, were determined in the hospital's laboratory department.

### Carotid Ultrasonography and Patient Classification Based on Plaque Stability

All subjects were given color ultrasonography examination of the carotid after admission. Specified criteria for atherosclerosis were as follows: IMT <1.0 mm was considered normal, 1.0 mm ≤ IMT <1.2 mm indicated the presence of carotid artery atherosclerosis, and IMT ≥ 1.2 mm was defined as plaque formation.

The stability of the plaque was determined according to the nature of echo revealed by utrasonography. The high-level echo indicated stable plaques, while the low-level echo or equal echo indicated unstable plaques. We divided patients into unstable plaque group and non-unstable plaque group. Specifically, the unstable plaque group included hypoechoic plaques, iso-echoic plaques and mixed plaques while non-unstable plaque group included no plaque formation and hyperechogenic (stable) plaques.

### Computed Tomography Angiography (CTA) and Patient Classification

CTA examination of the head and neck were performed to evaluate cerebral artery stenosis or occlusion. The following arterial segments were evaluated: Intracranial arteries included the intracranial segment of the internal carotid artery (ICA) and vertebral arteries (VA), the basilar artery (BA), the middle cerebral artery (M1, M2), the anterior cerebral artery (A1, A2), and the posterior cerebral artery (P1, P2). Extracranial arteries included the proximal ICA and proximal VA. Based on the degree of carotid artery stenosis (CAS) the study subjects were further divided into three groups according to the criteria of North American Symptomatic Carotid Endarterectomy Trial (NASCET) (15): the no to mild stenosis group (<50% stenosis), the moderate stenosis group (ranging from 50 to 69% stenosis), and the severe stenosis or occlusion group (ranging from 70 to 100% stenosis).

### Statistical Analysis

All statistical analyses were performed using the software program SPSS Statistics version 25. Continuous variables were expressed as the mean value ± standard deviation or medians with quartile ranges (QR) according to the normality of measured data distribution. Univariate comparison of two groups was performed with Unpaired *T*-test or Mann-Whitney U test. Categorical variables were presented as the percentages (%) of the subjects and the comparisons were performed using the Chi-Square test. Spearman rank correlation analysis was performed for the correlation analysis. Binary logistic regression analysis was performed to determine the independent risk factors for AIS. Receiver operating characteristic (ROC) analysis was performed to assess the diagnostic value of Lp-PLA2 in AIS and CAS. *P* < 0.05 in a two-tailed test was considered statistical significance.

## Results

**Figure 1:**
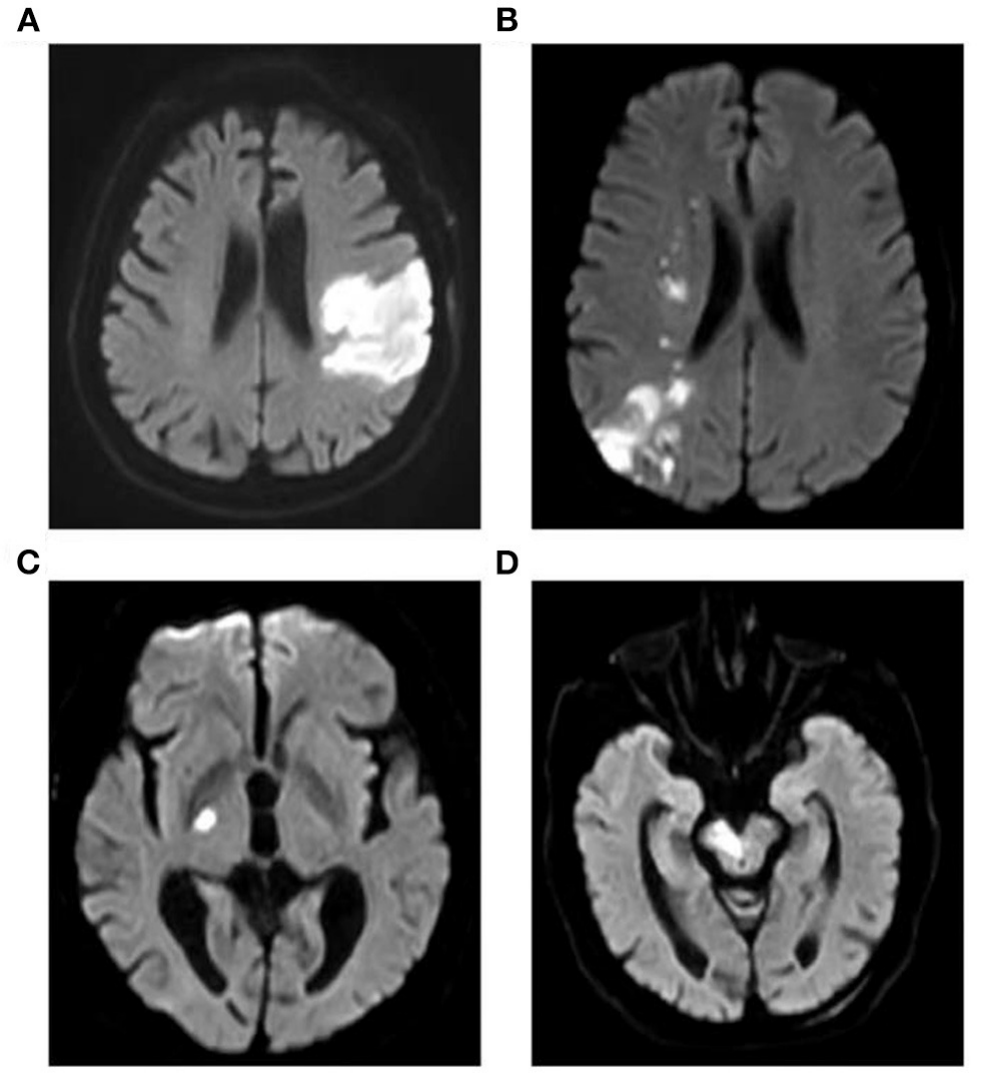
Brain MRI of AIS patients. Images show the typical manifestation of acute cerebral infarction on MRI. Bright hyperintensity displayed on DWI indicated a fresh infarct lesion. DWI demonstrated the infarction of the left temporal and occipital lobes **(A)** and a watershed infarction of the right hemisphere **(B)**. DWI showed the infarct of right hind limb **(C)** and brainstem **(D)**, respectively. MRI, magnetic resonance imaging; AIS, acute ischemic stroke; DWI, diffusion-weighted imaging.

A total of 200 AIS patients and 90 gender-and age-matched healthy controls were included in this study. The characteristic features of MRI of AIS patients were shown in Figure 1. Bright hyperintensity displayed on diffusion-weighted imaging (DWI) of MRI indicated a fresh infarct lesion. The demographic and clinical characteristics of all participants are shown in Table 1. There was no significant difference in the distribution of the gender, the age, smoking and alcohol consumption, and the blood level of CRE and UA between the AIS group and the control group. However, the following variables, including the percentage of people with hypertension or diabetes mellitus, and the levels of SBP, DBP, TC, TG, LDL-C, Glu were higher in the AIS group, whereas the level of HDL-C was higher in the controls. These differences were statistically significant (*P* < 0.05) (Table 1). Most significantly, the level of Lp-PLA2 was significantly higher in the AIS group than that in the control group (147.94 ± 41.48 vs. 88.75 ± 27.76, *p* = 0.001).

**Table 1:** Baseline characteristics of AIS patients and healthy controls.

**Characteristics**	**Healthy** ** controls** ** (*n* = 90)**	**AIS** ** patients** ** (*n =* 200)**	**Statistics**	***P***
Age (years)	64.18 ± 10.25	62.59 ± 9.54	1.285^a^	0.200
Male [*n* (%)]	50 (55.6)	129 (64.5)	2.102^b^	0.147
Hypertension [*n* (%)]	48 (53.3)	139 (69.5)	7.083^b^	0.008
SBP(mmHg)	138.69 ± 16.09	149.98 ± 22.00	−4.906^a^	0.001
DBP(mmHg)	81.11 ± 10.77	86.61 ± 13.26	−3.454^a^	0.001
Diabetes [*n* (%)]	10 (11.1)	59 (29.5)	11.576^b^	0.001
Smoking [*n* (%)]	20 (22.2)	63 (31.5)	3.152^b^	0.207
Alcohol drinking [*n* (%)]	18 (20.0)	55 (27.5)	2.370^b^	0.306
Glu (mmol/L)	5.08 (0.69)	5.33 (1.80)	−2.754^c^	0.006
CRE (umol/L)	62.00 ± 14.71	64.06 ± 14.82	−1.096^a^	0.274
UA (umol/L)	321.21 ± 76.20	316.96 ± 98.12	0.401^a^	0.689
TC (mmol/L)	4.00 ± 1.01	4.45 ± 0.94	−3.669^a^	0.001
TG (mmol/L)	1.23 (0.66)	1.39 (1.05)	−2.552^c^	0.011
HDL-C (mmol/L)	1.16 ± 0.25	1.05 ± 0.19	3.736^a^	0.001
LDL-C (mmol/L)	2.30 ± 0.87	2.75 ± 0.77	−4.447^a^	0.001
Lp-PLA2 (ng/ml)	88.75 ± 27.76	147.94 ± 41.48	−14.287^a^	0.001

*SBP, systolic blood pressure; DBP, diastolic blood pressure; Glu, fasting blood glucose; CRE, creatinine; UA, uric acid; TC, total cholesterol; TG, triacylglycerol; HDL-C, high-density lipoprotein cholesterol; LDL-C, low-density lipoprotein cholesterol; Lp-PLA2, lipoprotein-associated phospholipase A2*.

a *the t value obtained by the t-test*;

b *the value of χ2 obtained by the chi-square test*;

c *the Z value obtained by the Mann-Whitney U test*.

To examine the potential contribution of Lp-PLA2 to the occurrence of AIS in more details we divided AIS patients into severe stenosis or occlusion group (*n* = 96), moderate stenosis group (*n* = 41) and no/mild stenosis group (*n* = 63) based on the degree of cerebral vascular stenosis indicated by CTA. There were significant differences in serum Lp-PLA2 levels among these groups (Table 2), with the lowest level in the no/mild stenosis group (110.03 ± 22.68), followed by those in the moderate stenosis group (142.96 ± 16.34) and the severe stenosis or occlusion group (176.09 ± 34.70) (*P* < 0.005). Based on the results of carotid color ultrasonography, we also divided the AIS patients into the non-unstable plaque group (*n* = 96) and the unstable plaque group (*n* = 131). The level of Lp-PLA2 were significantly higher in the unstable plaque group than in the non-unstable plaque group (159.28 ± 41.34 vs. 128.01 ± 29.25, *p* = 0.001) (Table 2). These results indicated that a higher level of Lp-PLA2 was associated with a more severe atherosclerosis as reflected by the degree of carotid stenosis and the unstability of the carotid plaques.

**Table 2:** Comparison of serum Lp-PLA2 levels in patients with different degrees of cerebral vascular stenosis or plaque stability.

**Subgroup**	**No to mild stenosis**	**Moderate stenosis**	**Severe stenosis or occlusion**	**Unstable plaque**	**Non-unstable plaque**
N	63	41	96	131	69
Lp-PLA2 (ng/ml)	110.03 ± 22.68	142.96 ± 16.34	176.09 ± 34.70	159.28 ± 41.34	128.01 ± 29.25

*Lp-PLA2, Lipoprotein-associated phospholipase A2. There are statistical differences in the Lp-PLA2 levels between the following subgroups*.

*(1) No to mild stenosis group vs. moderate stenosis group, p < 0.001*.

*(2) Moderate stenosis group vs. severe stenosis or occlusion, p < 0.001*.

*(3) No to mild stenosis group vs. severe stenosis or occlusion group, p < 0.001*.

*(4) Unstable plaque group vs. non-unstable plaque group, p < 0.001*.

**Figure 2:**
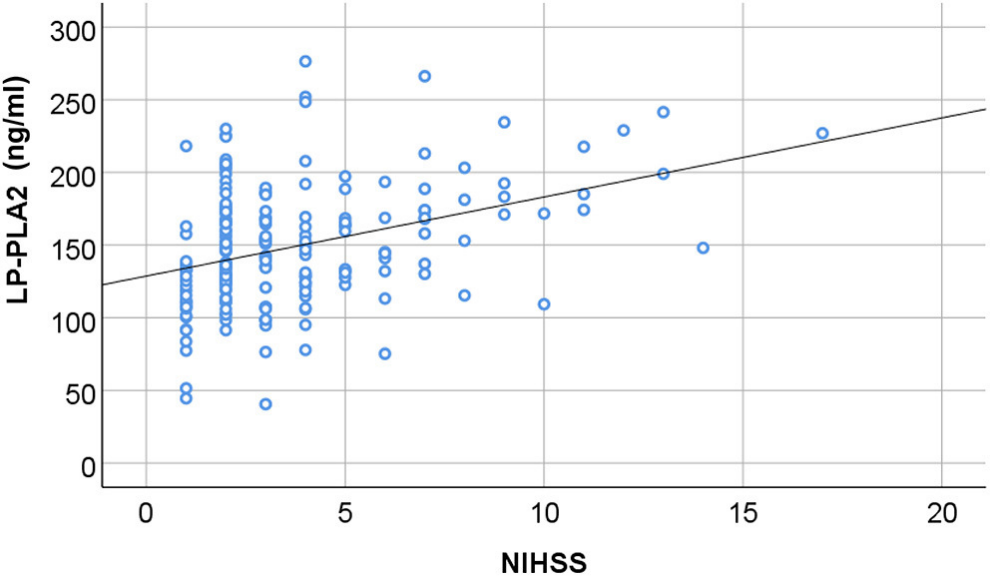
The correlation between serum Lp-PLA2 level and neurological impairment. The serum level of Lp-PLA2 (Lipoprotein-associated phospholipase A2; Lp-PLA2) in the AIS patients was plotted in association with the NIHSS scores at admission. There is positive correlation between the two parameters (*r* = 0.335, *P* = 0.001, by Spearman correlation analysis).

We would like then to determine whether the level of Lp-PLA2 could be correlated with the clinical severity of the AIS patients. According to the NIHSS score at admission, we divided AIS patients into the group with mild neurological injure (*n* = 109) and the group with moderate to severe neurological injury (*n* = 91). We found that the level of Lp-PLA2 was significantly higher in the moderate to severe group than in the mild group (169.61 ± 42.17 vs. 130.87 ± 28.88, *p* = 0.001) (Table 3). In addition, spearman correlation analysis showed that serum Lp-PLA2 level was positively correlated with NIHSS score at the admission in the AIS group (*r* = 0.335, *p* = 0.001) (Figure 2). These results indicate that a high Lp-PLA2 level can be a risk factor for the severe presentation of AIS.

**Table 3:** Correlation between serum Lp-PLA2 level and the severity of neurological impairment.

**Subgroup**	**Mild** ** neurological** ** impairment**	**Moderate-severe** ** neurological** ** impairment**	***t***	***p***
N	109	91	−7.431	0.0001
Lp-PLA2 (ng/ml)	130.87 ± 28.88	169.61 ± 42.17		

*Lp-PLA2, Lipoprotein-associated phospholipase A2*.

To further examine the risk factors for AIS, we performed binary logistic regression analysis. Without adjusting for any other confounding factors, the result suggested that the levels of Lp-PLA2 was significantly associated with the risk of AIS with the crude odds ratio (OR) of 1.053 [*p* = 0.001, 95% confidence interval (CI): 1.040–1.066]. Then we adjusted for the statistically significant indicators between the AIS group and the control group as listed in Table 1, which include the hypertension and the level of Glu, TC, TG, HDL-C, or LDL-C. The result showed that the levels of Lp-PLA2 was still significantly associated with the risk of AIS with the adjusted OR of 1.057 (*p* = 0.001, 95% CI: 1.040–1.075). Both results have a statistical significance with the *P*-value of 0.001, despite that the OR values were relatively small, indicating that Lp-PLA2 level could be independently related to the risk of AIS.

**Figure 3:**
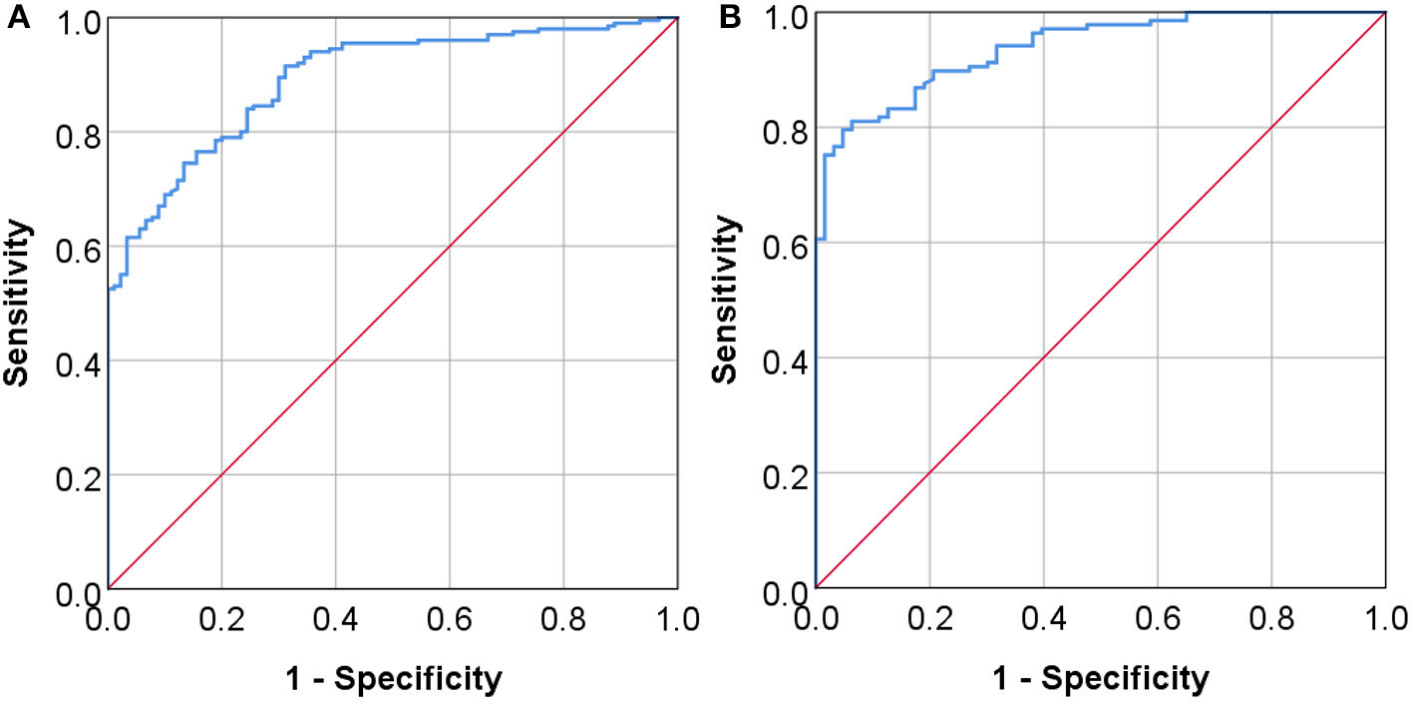
The diagnostic values of serum Lp-PLA2 levels. The ROC curves (receiver operating characteristic curve) of serum Lp-PLA2 level are constructed to assess its diagnostic values for AIS **(A)** and for the presence of moderate to severe stenosis or occlusion of the vessels **(B)**. The area under ROC curve (AUC) in panel A was 0.892 (*P* < 0.05, 95% CI: 0.856–0.929), and the AUC in **(B)** was 0.938 (*P* < 0.05, 95% CI: 0.908–0.968).

These findings suggest to us that Lp-PLA2 may server as a potential marker for AIS and stenosis. We thus determined the diagnostic value of the serum Lp-PLA2 level by constructing the ROC curve (Figure 3). We found that when the optimal serum level of Lp-PLA2 was set at 123.365 ng/ml, the sensitivity and specificity for the diagnosis of AIS using Lp-PLA2 were 74.5 and 86.7%, respectively, and the area under ROC curve (AUC) was 0.892 (*p* < 0.05, 95% CI: 0.856–0.929) (Figure 3A). Similarly, when the optimal serum level of Lp-PLA2 level was set at 136.46 ng/ml, the sensitivity and specificity for the diagnosis of stenosis at the moderate to severe degree or at the level of occlusion were 79.6 and 95.2%, respectively, and the AUC was 0.938 (*p* < 0.05, 95% CI: 0.908–0.968) (Figure 3B). These results thus suggest that Lp-PLA2 may be clinical useful as a potential marker for AIS and significant carotid stenosis.

## Discussion

At present, the diagnosis of AIS mainly depends on neural physical examination, CT scan, or MRI scan. CT has a low sensitivity and specificity for the diagnosis of AIS at the early stage. Although CT or MRI of the brain can be performed within 30 min of the onset, enabling diagnosis in the early stage of acute ischemic stroke, these imaging methods require experienced staff and sophisticated equipment that are not available in many local hospitals in developing countries such as China. Therefore, the search for effective blood biomarkers for assisting rapid diagnosis of AIS is an urgent clinical need. Unfortunately, there are still not many researches on this aspect. The determination of Lp-PLA2 as a potential biomarker in this study suggests that it may serve as a supplementary evaluation tool to the basic imaging-based diagnosis. Our study also provides additional insights into the pathophysiological mechanisms of AIS, which may help developing new therapeutics for ischemic cerebrovascular diseases.

Multiple studies have indicated that inflammatory response and various inflammatory factors play an important role in the development of AIS (16, 17). Lp-PLA2 is mainly released from atherosclerotic plaque by macrophages and neutrophils (18), which plays an important role in the process of atherosclerosis and participates in the development of plaque. Recent studies have confirmed that Lp-PLA2 is a new vascular-specific inflammatory factor that can be used as an independent risk factor for cardiovascular and cerebrovascular events (19, 20). However, its specific involvement in AIS and carotid artery stenosis was not clear.

Our study indicates that the elevated Lp-PLA2 level is not only strongly associated with ACI, but is also an independent risk factor for AIS, suggesting that serum Lp-PLA2 can be a potential early biomarker for the prediction and diagnosis of AIS. These findings are consistent with previous studies in which elevated levels of serum Lp-PLA2 were found in patients with stroke (21–23), and Lp-PLA2 can be an independent predictor of coronary heart disease and ischemic stroke in the general population (23). Furthermore, our study reveals that elevated Lp-PLA2 levels correlate with the incidence of plaque instability and cerebrovascular stenosis. This finding is also supported by other studies that have shown Lp-PLA2 was extensively expressed within areas of the necrotic core of advanced atherosclerotic lesions, colocalizing with apoptotic macrophages (24). It has also been shown that higher Lp-PLA2 levels may induce a series of inflammatory reactions (25–27). These and our studies together support the hypothesis that Lp-PLA2 and its products may play a role in promoting plaque instability and vascular stenosis, which trigger AIS.

Importantly, our results demonstrate that there is a positive correlation between Lp-PLA2 level and NIHSS score, and serum Lp-PLA2 levels can reflect the severity of neurological impairment. Previous studies have also found that Lp-PLA2 is independently related to admission severity and early neurological deterioration in ischemic stroke patients (28, 29). Thus, Lp-PLA2 level can be clinically important by helping therapeutic management. From this point, we have further investigated the utility of serum Lp-PLA2 level for the diagnosis of AIS and for the presence of severe cerebrovascular stenosis in these patients. The ROC curve indicates that serum Lp-PLA2 level has a high diagnostic value for AIS and severe stenosis.

The precise mechanism by which an elevated Lp-PLA2 level influences the occurrence and outcomes of AIS is not fully understood. Several potential pathophysiological mechanisms have been proposed, including inflammation, accelerated atherosclerosis or increased instability of atherosclerotic plaques (30, 31). Lp-PLA2 hydrolyzes oxidized phospholipids in LDL-C to produce lysophos-phatidylcholine (lysoPC), which has been found to be cytotoxic to vascular smooth muscle cells and can induce the local production of matrix metalloproteinases (MMPs). MMPs can thinner the fibrous cap and destabilize the architectural integrity of an atheromatous plaque, increasing its propensity to rupture (32). Furthermore, elevated Lp-PLA2 can increase the instability of atherosclerotic plaques by promoting inflammation and the formation of a necrotic core (33). Our study supports the relevance of the above mechanisms.

We recognize that this study has its limitations. First, our study was a retrospective study. Therefore, there could be selection bias. Second, serum Lp-PLA2 activity, the other important feature of Lp-PLA2, was not assessed in this study. It was reported that there was a modest correlation between the mass and activity of Lp-PLA2 (34). However, conflicting results from different studies for both mass and activity of Lp-PLA2 were also reported (21, 22). Further studies are needed to investigate the value of Lp-PLA2 activity in acute ischemic stroke. Thirdly, our study was done in one hospital, so it may not accurately reflect what happens in the general population. Hence, prospective multicenter clinical follow-up with larger sample sizes are needed to confirm that measurement of Lp-PLA2 can help to determine the risk of AIS. Further longitudinal study is needed to verify the causative relationship of Lp-PLA2 and AIS.

In conclusion, this is a thorough study demonstrating that serum Lp-PLA2 level can be a potential biomarker for the prediction and diagnosis of AIS. An elevated Lp-PLA2 level is also correlated with carotid plaque instability, severe neurological injury and cerebrovascular stenosis. Importantly, serum Lp-PLA2 level is valuable for helping the diagnosis of AIS and severe artery stenosis. These findings also provide a better basis for us to explore the mechanistic relationship between Lp-PLA2, carotid atherosclerotic plaque, and cerebral artery stenosis, and provide a new way for understanding the pathogenesis of AIS.

## Data Availability Statement

The raw data supporting the conclusions of this article will be made available by the authors, without undue reservation.

## Ethics Statement

The studies involving human participants were reviewed and approved by the Ethics Review Committee of the First Affiliated Hospital of Anhui Medical University. Written informed consent for participation was not required for this study in accordance with the national legislation and the institutional requirements.

## Author Contributions

JC: design and perform experiments, identification of case, analyze data, and write the manuscript. PY, YZ, and XZ: identification and analysis of cases. ZS: concept, discussion, and case. X-QZ: concept, experimental design, and write the manuscript. All authors contributed to the article and approved the submitted version.

## Conflict of Interest

The authors declare that the research was conducted in the absence of any commercial or financial relationships that could be construed as a potential conflict of interest.
